# Nanoparticle-Based Drug Delivery Systems Targeting Tumor Microenvironment for Cancer Immunotherapy Resistance: Current Advances and Applications

**DOI:** 10.3390/pharmaceutics14101990

**Published:** 2022-09-21

**Authors:** Peijie Wu, Jun Han, Yanju Gong, Chao Liu, Han Yu, Na Xie

**Affiliations:** 1 School of Basic Medical Sciences, Chengdu University of Traditional Chinese Medicine, Chengdu 610075, China; 2 State Key Laboratory of Biotherapy and Cancer Center, West China Hospital, and West China School of Basic Medical Sciences & Forensic Medicine, Sichuan University, and Collaborative Innovation Center for Biotherapy, Chengdu 610041, China

**Keywords:** immunotherapy, resistance, nanoparticles, tumor microenvironment, macrophages, fibroblasts, tumor vasculature, hypoxia, oxidative stress

## Abstract

Cancer immunotherapy has shown impressive anti-tumor activity in patients with advanced and early-stage malignant tumors, thus improving long-term survival. However, current cancer immunotherapy is limited by barriers such as low tumor specificity, poor response rate, and systemic toxicities, which result in the development of primary, adaptive, or acquired resistance. Immunotherapy resistance has complex mechanisms that depend on the interaction between tumor cells and the tumor microenvironment (TME). Therefore, targeting TME has recently received attention as a feasibility strategy for re-sensitizing resistant neoplastic niches to existing cancer immunotherapy. With the development of nanotechnology, nanoplatforms possess outstanding features, including high loading capacity, tunable porosity, and specific targeting to the desired locus. Therefore, nanoplatforms can significantly improve the effectiveness of immunotherapy while reducing its toxic and side effects on non-target cells that receive intense attention in cancer immunotherapy. This review explores the mechanisms of tumor microenvironment reprogramming in immunotherapy resistance, including TAMs, CAFs, vasculature, and hypoxia. We also examined whether the application of nano-drugs combined with current regimens is improving immunotherapy clinical outcomes in solid tumors.

## 1. Introduction

Cancer has been a significant and escalating public health issue, which caused one in six deaths worldwide [1]. It has been reported that 19.3 million new patients and 10.0 million deaths associated with cancer were counted in 2020 [2]. However, the numbers are still increasing; more than 29.4 million cases of cancer may be estimated 20 years later [3]. Accordingly, efforts have been made to eliminate cancer through several strategies, including surgery, radiotherapy, chemotherapy, targeted therapy, and immunotherapy [4].

**Figure 1:**
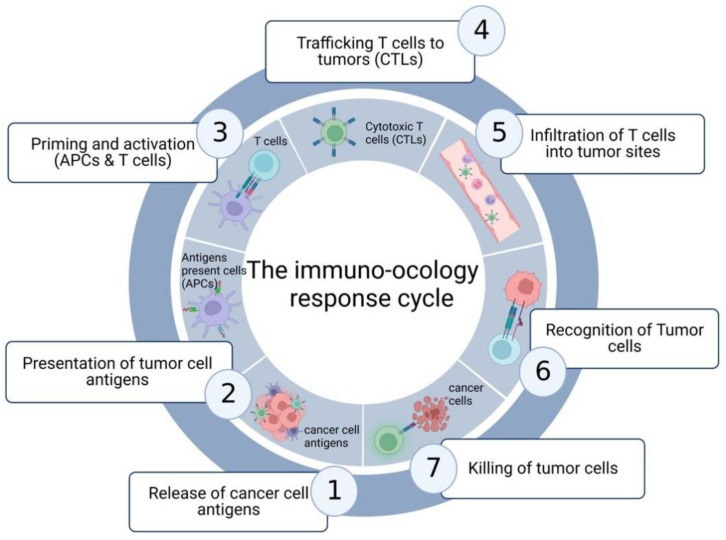
The response of the immune system to cancer cells. This process is composed of seven steps, including the release of cancer cell antigens, presentation of tumor cells antigens, T cells priming and activation by APCs, T cell trafficking, infiltration of cytotoxic T cells (CTLs) into tumor sites, recognition of tumor cells and the killing of cancer cells by CTLs.

Recently, tumor immunotherapy has been demonstrated to be a powerful and novel therapeutic regimen with great durability and specificity against cancers [5]. Immunotherapy is research based on the communication between cancer and the host immune system [6]. Actually, the hypothesis of tumor immunosurveillance was firstly proposed by Burnet and Thomas in 1957 [7,8]. They found that immune cells could recognize tumor cells at the early stages and kill them by secreting interferon-γ (IFN-γ). Additionally, tumor immunotherapy has been recognized since early 1990 in pre-clinical and clinical trials with immune-stimulating cytokines, cancer vaccines, and adoptive T cell therapy. Intriguingly, recent studies have demonstrated that both radiotherapy and chemotherapy could regulate the response of the immune system to cancer cells in recent years (Figure 1) [9]. Accordingly, many efforts have been made to develop immunotherapy by targeting the interactions between tumor and immune systems [8]. Several substantial advances have been made in various immunotherapies as new paradigms against cancer, such as the immune checkpoints inhibitors, administration of specific cytokines, CAR-T and CAR-NK. These advances could be mainly summarized as the modulation of immune cell activity via T cells by using antibodies and adoptive cell delivery [10,11]. Among these, immune checkpoint inhibitors may be the widest and most effective immunotherapy for a various solid tumors and hematologic malignancies to date. Immune checkpoint inhibitors are used to enhance the immune response using antibodies targeting PD-1 or CTLA4 on the cellular membrane of tumor cells and T cells [12]. Therefore, there has been a rapid increase in interest in immunotherapy, such as the research on the immune escape during the treatment of cetuximab and early clinical studies of immune checkpoint inhibitors [13]. These have been implicated in successful results not only for malignant melanoma but also for non-small-cell lung cancer as well as head and neck cancer [14]. For example, the latest study suggested that a combination of anti-CTLA4 and anti-PD1 against metastatic melanoma and non-small-cell lung carcinoma (NSCLC) increased the patient’s life expectancy and inhibited metastasis [15,16].

Although cancer immunotherapy is a novel and attractive regimen, it is also limited by some barriers, including low immunogenicity of tumor cells, the high immunosuppression of the tumor microenvironment, and systemic toxicities [17]. The tumor microenvironment (TME) remains the main barrier to cancer therapeutic intervention. The features of the tumor microenvironment may be complex due to their heterogeneity, in accordance with their immune response, proliferation, and metastasis. The TME, besides tumor cells, also includes a variety of other cell types such as T cells, vasculature, extracellular matrix (ECM), macrophages, fibroblasts, chemokines, and hypoxia (Table 1) [18,19,20]. Furthermore, tumor cells could affect immune systems by constructing an immunosuppressive TME to promote tumor cell growth and immune evasion. In fact, recent evidence has indicated that various cancers would diminish normal immune capacity against tumor cells leading to immunoediting processes that facilitate tumor growth and redistribution at the later stages, such as increasing tumor-associated macrophages (TAMs) and regulatory T cells, decreasing absolute counts of lymphocytes, and apoptosis of cytotoxic T cells [9,21,22]. Furthermore, some components existing in the tumor microenvironment were also found to directly reduce the immune response against cancer, including prostaglandin E2, vascular endothelial growth factor, interleukin IL-10 secreted from cancer cells, and transforming growth factor-β (TGF-β) [23,24,25,26]. Many researchers also demonstrated that tumor cells could hijack the host immune system to promote tumor development, such as the inhibition of Th1 cells, which induces cytotoxic T cell differentiation, and the antagonistic Th2 response [27]. Cancer immunotherapy also needs to overcome not only the immune checkpoints but also the immunosuppressive TME [20,28,29]. Therefore, targeting TME has received attention as a potential method for re-sensitizing resistant tumor cells to existing therapy. It is important to elevate our comprehensive understanding of the interaction between tumor cells and the TME leading to immunotherapy resistance [30,31].

**Figure 2:**
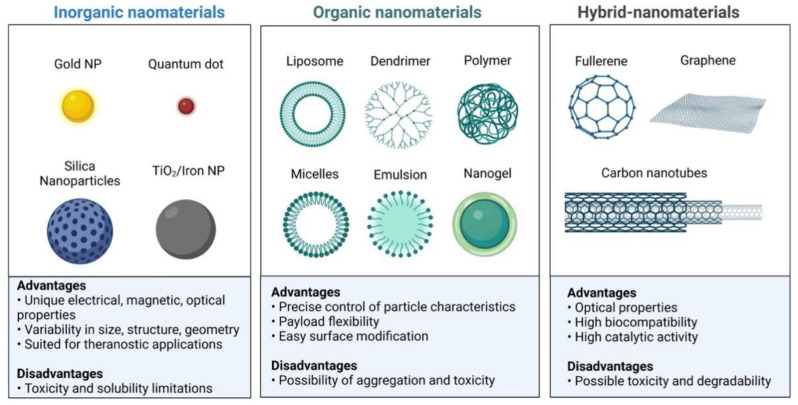
Nanoparticles have been demonstrated to possess great advantages including abundant surface modification, tunable structures, high loading capacity of biomolecules, and controllable release molecules.

In addition, caution is also needed to ensure that tumor immunotherapy only targets specific tumor tissue to inhibit side effects such as systemic toxicity. Although the mechanisms of TME reprogramming have been studied for long enough to be considered the basis of cancer therapy resistance, active research is still undergoing, and targeting TME remains to be an effective and promising strategy [50,51]. Therefore, various specific aspects of targeting TME are noteworthy for future cancer research [51]. Interestingly, Nanoparticle-based drug delivery systems provide new tools for these issues, receiving intense attention in tumor immunotherapy due to their unique biological and chemical properties [52]. Porous nanomaterials (NPs) have been widely used in several biomedical fields with high loading capacity and tunable porosity, especially as drug carriers [53,54]. NPs have been demonstrated to possess great advantages including abundant surface modification, tunable structures, high loading capacity of biomolecules, and controllable release molecules (Figure 2) [55,56,57]. In fact, NP-based drug delivery systems were also found to be effective to enhance tumor immunotherapy and reduce immune-related adverse events through several methods including modulating immune dysfunction in TME, specific tissue or organ targeting, and delivering drugs into target cells/tissues [58,59]. Moreover, PNMs also can be modified to combine tumor immunotherapy with other treatments to achieve better clinical effects [60,61,62].

In this review, we summarize the roles of the tumor microenvironment in immunotherapy resistance, including TAMs, CAFs, vasculature, and hypoxia. We also examined the recent progress of employing nano-drugs targeting TME on improving immunotherapy clinical outcomes in solid tumors, such as targeting immunosuppressive components, targeting cancer-associated fibroblasts, targeting the tumor vasculature, and targeting tumor chemical or physiology in TME. Finally, we discussed the relevant challenges and future directions of applying NPs for cancer immunotherapy that urgently need to be addressed in clinical practice, coupled with corresponding solutions to these problems.

## 2. The Advantages of Nanoparticle Strategies Targeting the Tumor Microenvironment for Cancer Immunotherapy Resistance

Nanoparticle strategies have attracted much interest in cancer therapy due to the following properties: low cell toxicity, favorable pharmacokinetics and pharmacodynamics, outstanding biocompatibility, preeminent mechanical and chemical robustness, great permeation and retention (EPR) effects, etc. [59,63,64]. In addition, nanoparticles (NPs) could overcome the challenges of traditional drug carries and provide novel regimens to trigger stronger immune responses. Thus, NPs have received more attention as a potential strategy for promoting immunotherapies owing to their extended retention time as well as cell/tissue specificity [65]. The tumor microenvironment (TME) of solid tumors remains one of the main barriers to successful therapeutic intervention in cancer treatment. The tumor microenvironment (TME), besides tumor cells, also contains a variety of normal cells such as macrophages, NK cells, T cells, fibroblasts, dendritic cells, and adipocytes [18]. Therefore, these complex components of the tumor microenvironment remain an issue that impedes tumor immunotherapy. However, NPs may be versatile, effective, and appropriate agents targeting TME to combine with tumor immunotherapy. For instance, tumor growth and angiogenesis could active MDSCs and Tregs, secreting VEGF and TGF-β, which result in hypoxia and immunosuppression [66]. Recent studies demonstrated that several nanoparticles can selectively target these cells and molecules to reprogram the immunosuppressive environment towards a normal immune system and re-sensitize tumor cells to therapy. Some NPs also were reported to depose in tumor tissue compared with normal cells which depend on the aberrant lymphatic drainage, increased EPR effects, and leaky vasculature. In addition, NPs can be modified easily to conjugate with specific ligands to enhance compatibility, versatility, and efficiency [67]. Based on the nature of the materials, the NPs can be divided into three groups and the typical examples, advantages, and disadvantages are summarized in Table 2.

**Figure 3:**
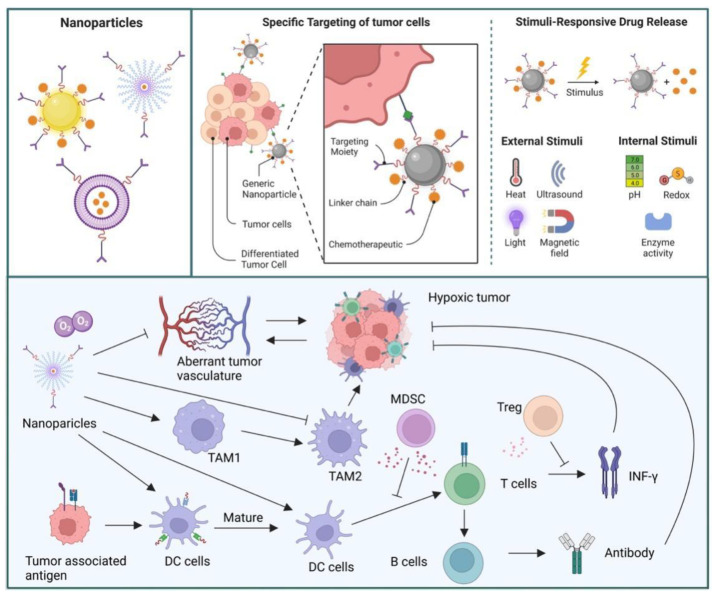
Nanoparticles have many properties which could release drugs by external or internal stimulation. These NPs also can inhibit tumor proliferation and progress by targeting TME. Nanomaterials can attenuate tumor resistance and hypoxia by targeting the aberrant vasculature. Moreover, NPs could promote macrophage shifts from M 2 into M 1 subtypes, which reactivate the immune system. Nanomaterials promote dendritic cell activation, causing the maturation of T cells and eradication of tumor cells.

We also summarized recent NPs (Table 3) targeting TME through different TME components to reprogram the immunosuppressive system and re-sensitize tumor cells to immunotherapy, such as targeting CAFs, TAMs, tumor extracellular matrix, and vasculature and immunosuppressive components (Figure 3).

## 3. Using Nanoparticle-Based Drug Delivery Systems to Target Cancer-Associated Fibroblasts (CAFs)

The tumor stroma mainly consists of fibroblasts and tumor cells and regulate tumor growth and development through reciprocal interaction [94]. Fibroblasts, also known as CAFs in cancer tissue, promote tissue repair healthy tissue through depositing the ECM [95]. Recent research has implicated that CAFs could promote drug resistance and obstruct drug delivery by enhancing the secretion α-SMA, vascular endothelial growth factor, and pro-angiogenic molecules [94]. Therefore, targeting these cells may be a key strategy to reprogram the tumor environment and inhibit immune escape. For example, there have been two types of CAF-based nanoparticle strategies: (1) disruption of the barriers related to CAFs and (2) targeting CAFs to enhance therapeutic efficacy against cancer [94].

### 3.1. Disruption of the Barriers Related to CAFs

(1) Targeting myofibroblasts, one of the important components of CAFs [94]. The formation of myofibroblasts is dependent on TGF-β1, which enhances ROS and α-SMA levels, and is inhibited by anti-oxidants [96]. For example, Alili et al. have found that nanoceria can regulate myofibroblast formation, reduce α-SMA myo-fibroblastic cells, and inhibit the invasion of tumor cells [97]. These results indicated that they may be an effective and safe treatment strategy [97].

(2) Targeting pancreatic stellate cells, the primary cancer-associated fibroblast precursors in tumor stroma. For instance, recent studies developed super-paramagnetic iron oxide nanomaterials improved by relaxin-2, which inhibits the differentiation of pancreatic stellate cells by inhibiting the Smad2 signaling [98]. The relaxin-2 NPs were found to promote targeted drug delivery and suppressed collagen deposition. These results demonstrated that NP strategies could enhance stroma modulation and improve drug pharmacokinetics. Moreover, other researchers designed injectable hydrogel nanoparticles carrying losartan, which enhanced encapsulation through self-arrangement [99]. It was successfully retained for several days and inhibited both collagen and cancer-associated fibroblast levels in orthotopic mice. Chen et al. reported codelivery and pH-sensitive NP systems composed of a p-GEM and paclitaxel NP [100]. These systems could target the deeper stroma while delivering drugs. They demonstrated that the NPs specifically reduced the α-SMA level in tumor tissue and destroyed cancer cells without regulating another stroma [100]. Accordingly, these NPs showed properties with higher drug delivery efficiency, specific targeting, and warranted further research to achieve the therapeutic outcomes.

### 3.2. Targeting CAFs to Elevate the Efficacy of Tumor Treatment

(1) Establish physical barriers to impede drug absorption and penetration [94]. Recent studies have shown that Wnt16, increased in cis-treated CAFs, results in cisplatinum resistance [101]. Cisplatinum upregulated Wnt16 expression, causing stroma reconstruction and enhancing therapy resistance. However, the nanoparticles carrying wnt16-siRNAs were confirmed to promote cisplatinum cytotoxicity within the stroma-abundant environment [101]. These NPs could downregulate Wnt16 to elicit several advantages, such as re-sensitizing tumor cells to cisplatinum, reprogramming the TME, as well as inhibiting angiogenesis [101]. Another study also indicated the therapeutic effects of NPs carrying quercetin which could reduce the Wnt16 level in CAFs [102]. The NPs significantly decreased Wnt16 expression and re-sensitized tumor cells to cisplatin in stroma-enriched bladder cancer. These two studies indicate that CAF targeting and NP re-sensitization in tumors can reprogram the TME and enhance cytotoxicity.

(2) Targeting and modifying CAFs with NPs. Huang et al. designed nanomaterials carrying plasmids expressing sTRAIL and administered to mouse xenograft models. They proved that these NPs were effective in a xenograft PC model by inducing apoptotic death of CAFs and reprogrammed CAFs to a quiescent state. They also found that these NPs could ablate the tumor and remodel the TME to promote the second therapeutic treatment. Another study also developed CAF-targeting NPs carrying navitoclax, which would selectively combine to the tenascin C [103]. These NPs were reported to improve cellular uptake and enhance cytotoxic effects. In addition, these NPs eliminated CAFs and inhibited cancer progression both in vitro and in vivo.

As these examples show, the NPs could reverse drug resistance and improve T cell infiltration and reshape the TME to reactivate anti-cancer pathways by selectively targeting CAFs. These elegant approaches could pave the way for personalized therapies and would combine with the latest therapeutic strategies to elevate clinical outcomes.

## 4. Nanoparticle Strategies for Targeting TAMs

Macrophages have been reported to play a crucial function in wound healing and tissue regeneration, immunity, and homeostasis [95,104]. Macrophages could differentially transform into M 1 and M 2 types depending on the stimulants. For instance, IFN-γ and lipopolysaccharide could induce classical M 1 subtype polarization, which impedes tumor cell growth by secreting IL-12. Exposure to IL-4 and IL-13 polarizes macrophages into M 2 types that promote tumor cell progression, tissue repair, and healing by increasing the expression of IL-12 [95]. According to these findings, macrophages are thought to be a double-edged sword. TAMs may promote tumor immunotherapy on tumor initiation but cause angiogenesis and become centers of immunodepression at an advanced stage, which may be dependent on the TME alterations [105]. Thus, specifically targeting TAMs may be a promising cancer immunotherapy strategy.

### 4.1. Regulating TAM Polarization

(1) Regulating TAM polarization. Recent studies proved that iron-oxide nanoparticles such as ferumoxytol, recently approved for the symptom of iron deficiency, could promote polarization of macrophages from M 2 towards M 1 types, inhibiting tumor cell growth [106]. Another iron-based NP can change TAM-binding molecules on S dots, which carried many O_2_-containing groups on the surface [107]. Once internalized by lysosomes, these NPs released irons and induced ROS, thus transforming macrophages from M 2 towards M 1. Moreover, NPs of ultra-tiny size promoted deeper cancer issue penetration [107]. These studies implicated that the endogenous components may be utilized to overcome therapy resistance.

Other groups constructed a biomimetic formula against tumors to reprogram M 2 subtypes and elevate cancer immunotherapy, which coated resiquimod (R848) with PLGA and B16-OVA membrane [108]. These nanomaterials could upregulate the expression of CD47 that would protect the nanomaterials from clearance [108]. Hence, this nano-based strategy, targeting Toll-like receptors, may offer a promising, selective, and efficient method for re-polarizing M 2 subtype TAMs. Moreover, other groups have developed several alternative approaches to enhance macrophage reprogramming.

(2) Tumor vaccine based on TMPs (tumor-derived antigenic microparticles). Advanced nano-based approaches, TMPs, have similar cytosolic components and biological properties with relevant parentals [109]. T-MPs display universal immunogenicity and are hopeful vehicles due to the highly immunogenic antigens and are exposure free [109]. For instance, some studies reported an effective tumor vaccine with TMPs that has shown effective anti-cancer effects in several solid tumors. They loaded nano-Fe_3_O_4_ TMPs with abundant CpG/Lipo on surfaces to produce a vaccine-pooled cancer antigen, and immune-stimulant/modulator. This novel vaccine was proved to re-polarized macrophages towards M 1 subtypes and increased cytotoxic T cell (CTL) numbers [109]. Some studies also reported that nanoparticles covered with NK cellular membranes, can promote M 1 polarization and enhance anti-cancer responses [110]. Wu et al. developed pFEOOH-NRs using FeOOH nanorods with PAA [111]. They found that the pFEOOH-DOX loaded nanoparticles could be applied for cancer clinical treatment by recovering the immunostimulant environment and causing tumor recurrence abolishment [111].

### 4.2. Suppression of TAM Survival and Function

To test this strategy, recent studies reported novel NPs with dual-targeting M 2-like properties and TAMs [112]. The function of these NPs was regulated by SRB1, combining with the peptide that specifically recognized M 2 subtype macrophages. These NPs displayed high selectivity extinction of M 2 macrophages, causing subsequent eradication of cancer cells. These dual-targeting NPs also decreased the levels of immune inhibitor molecules, such as TGF-β and IL-10, and upregulated IL-12 and TNF-α, promoted CTL infiltration, and recovered normal immune sensitivity [112]. Some studies also reported a similar strategy to enhance Ibrutinib (IBR), a chemical inhibitory drug of BTKs (bruton tyrosine kinase), uptake by TAMs [113]. These NPs were demonstrated to prolong IBR retention time and promote drug delivery, contributing to immune restoration and tumor suppression. These NPs (SA/IBR/EPG) modified with stearic acid may efficiently deliver IBR into macrophages and blocked BTK activation, resulting in the decrease in tumor volume and angiogenesis [113]. According to these findings, these NPs may decrease elimination and enhance the retention time and delivery by specific targets in the TME, leading to satisfactory immunotherapeutic outcomes. Therefore, these NP strategies provide a valuable clinical application that overcomes the traditional disadvantages including defective solubility, poor circulation, and non-specific delivery. However, these NPs need to be tested in the clinical trials to evaluate the practical therapies due to the complicated mechanisms of TAM polarization depending on the TME context.

## 5. Nanoparticle-Based Drug Delivery Systems for Modulating the Tumor Extracellular Matrix

**Figure 4:**
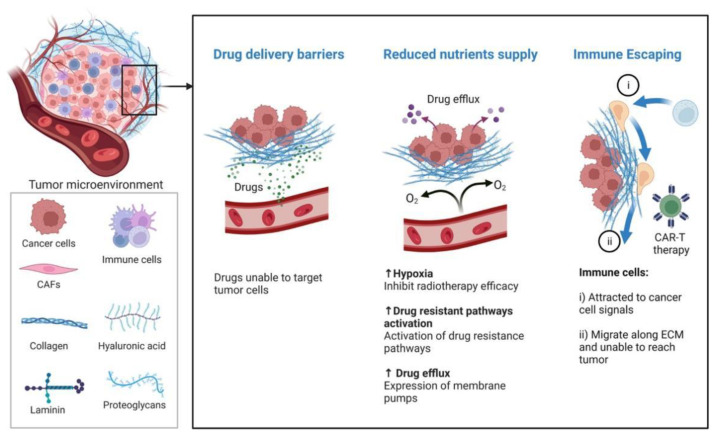
The ECM was found to offer structural support and regulate cellular functions, such as proliferation, communication, and adhesion (laminin, elastin, and collagen). Small molecular medicines are usually transported from interstitial areas towards tumor cells by the blood pressure. Mechanically, the organization of the ECM increases fluid pressure to inhibit drug delivery in interstitial spaces. For example, the extracellular matrix proteoglycan could upregulate inflammatory cytokines such as TNFα, IL-6, and NF-κB, to escape immune surveillance.

The ECM was found to provide structural support and regulate cellular activities, including proliferation, communication and adhesion, including laminin, elastin, and collagen (Figure 4) [114,115,116,117,118]. Additionally, the characters of the extracellular matrix are diverse, due to the resident cells, tumor tissue, and staging [119,120,121,122,123]. In general, the ECM contributes to tumor therapy resistance through promoting the escape from immune surveillance and inhibiting drug delivery. Small molecular medicines are usually transported from interstitial areas towards tumor cells by the blood pressure. Mechanically, the organization of the ECM increases fluid pressure to inhibit drug delivery in interstitial spaces [120,124]. In fact, these drugs must cross the ECM to achieve functions during tumor treatment. Moreover, drug delivery was significantly suppressed in the three-dimensional cultured spheroids compared with the two-dimensional monolayer due to the number of ECM components [121]. Some studies found that cancer cells acquired obvious resistance to 5-fluorouracil/oxaliplatin in collagen I matrix [121,125,126]. In addition, the extracellular matrix proteoglycan could upregulate inflammatory cytokines such as TNFα, IL-6, and NF-κB, to escape immune surveillance [39,40,41,42]. Studies suggested that the ECM, which plays an important role in tumor development, can be utilized for tumor therapy.

Recent research has been focused on targeting the ECM. For example, laminin has been reported to be one of the main components of the ECM. Some studies reported a laminin-mimicking peptide, which could self-assemble and form NPs through hydrophobic interactions, effectively inhibiting tumor invasion [127]. These NPs may be modified by laminin receptors or integrins in vivo, which could prolong retention time and accumulate at tumor sites. They were proved to successfully inhibit metastasis in multiple cancers [127]. Another approach was reported to use cell adhesion molecules that mimic ECM loading to assemblies of magnetic nanocarrier agarose hydrogels [128]. These magnetic NPs assembled into both mesh-like or fibrous patterns to guide metastasis driven by magnetostatic fields [128]. As these studies suggested, these NP strategies offer an innovative solution to modulate adhesion and metastasis by enhancing the ECM strength.

Other studies focus on different deposition stages of ECM to recover abnormal structures. For instance, some studies found LOXL-2-antibodie-loaded NPs, which would be able to alter the endogenic collagens without changing ECM components [129]. These NPs effectively destroyed cell adhesion and metastasis in various cancers by modifying collagen morphologies. These studies indicated that these NPs used to modify ECM architecture effectively eradicate tumor cells by shifting the tumor environment towards non-metastatic phenotypes. Meanwhile, hyaluronic acid (HA), a crucial component of the ECM, is a hydrophilic glucosamine polysaccharide that is mainly over-expressed in malignant tumor sites. HA has been found to play an important role in metastasis and growth of cancer cells by inhibiting the infiltration of immune cell tumor perfusion, while elevating tumor vascular collapse [130]. Thus, NPs based on HA have been applied to improve immunotherapy. For example, PLGA-PEG-rHuPH2 NPs were developed to elevate the efficiency and specificity of drug delivery via HAase activity [131]. Interestingly, collagen is one major components of the ECM in malignant tumor TMEs. Over-expression of collagen could cause therapy resistance and inhibit drug absorption [132]. Similar to HA degradation, NPs combined with collagen depletion have been developed to promote drug transport. For instance, the PLGA NPs were designed to improve drug release and treatment efficacy, which were composed of collagenase and doxorubicin (DOX) [133]. In addition, other groups have developed a thermosensitive PLGA-PEG-PLGA hydrogel carrying collagenase and trastuzumab to enhance immunotherapy [134]. However, NP-based collagenase used in tumor therapy may be much less common than NP-based HAase due to the less separated capacity of cleavage [135]. Among them, MMPs (matrix metalloproteinases) are another crucial component of the ECM, which was associated with tumor progress and development. For instance, recent studies have reported an NP based on MMP-9-cleavable lipopeptide to control tumor proliferation and enhance drug accumulation in the tumor site [136].

Accordingly, these NPs targeting ECM strategies open new insights of cancer treatment and attend promising possibilities of personalized therapy.

## 6. Nanoparticle-Based Drug Delivery Systems for Targeting the Tumor Vasculature

**Figure 5:**
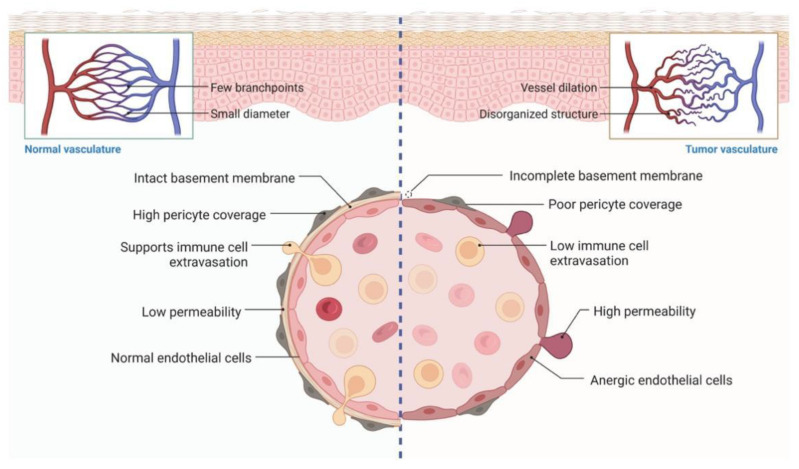
The vasculature usually transports nutrients, O_2_, growth factors, and waste products, which play an important role in tumor relapse, metastasis, and resistance. The vasculature was found to be complex, extended, branched, and have more loops in tumor tissue. The accumulation of metabolic waste and insufficient O_2_ can promote acidic and hypoxic environments, which contribute to drug resistance.

The vasculature could transport nutrients, O_2_, growth factors, and waste products, which play an important role in tumor relapse, metastasis, and resistance (Figure 5). Studies have demonstrated that the outcome of tumor treatment is influenced by the vasculature through drug delivery and the supply of nutrients and O_2_ [137]. The vasculature was found to be complex, extended, branched, and have more loops in tumor tissue [138]. The blood flow usually is chaotic and variable in tumor tissue, due to the discontinuous vessel walls and leakiness [139,140,141,142,143]. Moreover, the blood displayed high geometric resistance and viscosity for blood, and had low pressure between venules and arterioles in tumor tissues [143,144]. Recent studies also found that lymph vessels are absent or scarce in various solid tumors, leading to high interstitial pressure [44,143,145,146]. As a result, the large bio-molecules were significantly impeded and transported far away from tumor tissue [43,44]. In addition, the abnormal vasculature impairs the transportation of nutrition and O_2_ by decreasing blood flow due to the tumor cells’ excessive growth [147]. According to these findings, the accumulation of metabolic waste and ab insufficient amount of O_2_ promotes an acidic and hypoxic environment, which contributes to drug resistance [148]. Many studies have proved that the distribution of drugs is related to the distance from vascular systems to tumor tissues which play an important role in the outcomes of lung, breast, and liver cancers [143,149,150]. For example, the synergistic treatment with anti-angiogenic agents and a VEGFR inhibitor showed more efficient clinical outcomes compared with a single treatment alone [151]. It suggested that the resistance of the VEGFR inhibitor is related to proangiogenic factors. Moreover, growing evidence indicates that various of cytokines can regulate the generation of vasculature. For instance, CXCR7 have been proved to promote angiogenesis by promoting ERK1/2 phosphorylation [152]. Additionally, the complex of CXCL12 and CXCR7 was reported to promote lung metastasis and resistance via pro-angiogenesis [152,153]. The feature of tumor vasculature is aberrant functions and appearance. The hypoxic TME promotes the expression of angiogenic molecules, including TGF-β and VEGF, which impaired its activity. In fact, the imbalance between pro- and anti-angiogenic factors caused tortuous, unevenly distributed vessel generation in tumor niches [95]. The characteristics of these abnormal vessels are high permeability, resulting in protein leakiness and increased interstitial fluid pressure and exacerbates tumor hypoxia in the TME [95]. Finally, the dysregulated vessels could inhibit T cell infiltration and impair drug delivery, to generate phylactic barriers which protects the cancers and hampers clinical trials [95]. Thus, targeting the tumor vasculature may be an important strategy to restore the tumor immune responses.

Although targeting the vasculature may be a theoretical therapeutic strategy, its actual result has been obstructed by acquired endothelial resistance [95,154]. NPs carrying anti-angiogenic drugs have recently emerged as a potential strategy to target the vasculature, obviating endothelial resistance [95]. Recently, Du et al. designed a lipid-nanomaterial strategy that can normalize tumor vasculature and improve therapeutic responses, composed of drugs against angiogenesis, coupled with GEM and LMWH (small molecular weight heparin) [155]. Other groups developed an NP platform to facilitate tumor vasculature normalization using gold NPs carrying recombinant endostatin to reduce VEGF, which effectively inhibits angiogenesis and metastasis [156]. This platform was reported to reduce hypoxia, promote vessel normalization, enhance the accumulation of endostatin, and improve therapeutic responses in xenografts [156]. According to these findings, NPs could overcome therapeutic challenges by both targeting of the tumor vasculature and ECM and can improve the efficacy of nanotherapeutics [157].

Some studies also reported that near infrared (NIR)-laser-induced NPs were developed as a non-invasive strategy for destroying abnormal vasculature in a precise and rapid fashion [158]. After NIR radiation, many bubbles may be released expeditiously to disrupt the tumor vasculature and induce tumor cell necrosis, due to increased local temperatures [158]. Furthermore, nano-based vasculature-disrupting strategies might be efficient in synergy with immunotherapy. For example, Satterlee et al. used NPs to elicit apoptotic death in the hub while promoting NP absorption through VDAs to improve the accumulation of radiotoxicity [159]. Interestingly, the combination of VDAs with immunotherapy significantly reduced tumor volume and disrupted the activity of tumor vasculature. For example, other groups used TLR7/8 agonist encapsulated PLGA as platforms with gardiquimod in cooperation with the VDA, which significantly reduced melanoma tumor volume [160]. Thus, the synergy of VDA- and TLR-agonists would be one of effective approaches to prevent cancers. In addition, Zhou et al. combined anti-angiogenic drugs and immunotherapy by using antiangiogenic copper chelating polymers to fabricate NPs loaded with resiquimod [161]. This strategy dramatically inhibits tumor cell growth and metastasis in breast cancer through copper-deficiency-induced anti-angiogenesis and resiquimod-elicited immune activation [161]. Another group developed NPs with co-delivering copper (I) chelators and anti-angiogenic drugs [162]. The synthesized NPs were loaded with both doxorubicin and Probe, which would capture Cu^+^ through photoacoustic and NIR signals [162]. This platform successfully inhibited cancer progression without toxicity and offered possibilities of novel nanoparticles that may overcome the impediment of traditional therapies.

Accordingly, nano-based approaches incorporating immunotherapy with anti-angiogenic drugs warrant further development and investigation with high feasibility and efficiency.

## 7. Using Nanoparticle-Based Drug Delivery Systems to Target Immunosuppressive Components

### 7.1. Nanoparticles Targeting Immunosuppressive Components

The tumor–immunity cycle was reported to have four steps and starts with the secretion of antigens from tumor cells. Then, antigen-presenting cells (APCs) could recognize these antigens and promote naïve Treg cells to transform CTLs. The cytotoxic T cells identify and eliminate cancer cells by secretion of cytotoxicity components, such as perforin and granzyme B. This original cycle could trigger more secretion of tumor antigens and the activation of a second cascade [163]. Interestingly, the synergy of immunotherapy and nanoparticles targeting immunosuppressive components has been valued in a variety of clinical and pre-clinical outcomes. For instance, recent studies have reported a strategy through loading chemotherapeutic or photothermal agents into nanoparticles, that may effectively elicit immunogenic cell death (ICD) by inducing the antigen release [164]. Actually, nanoparticles carrying oxaliplatin promoted apoptosis and improved the immune response compared to free oxaliplatin in a prostate cancer model. Moreover, these NPs also promoted T cell infiltration, INF-γ secretion, and DC maturation [165]. Therefore, these studies indicated the therapeutic efficacy of oxaliplatin nanoparticles targeting immunosuppressive components. In addition, the NPs carrying antigens or adjuvants have been devised as a tumor vaccine to re-active immunoreaction by targeting antigens arising from somatic mutations in solid tumors [166]. Furthermore, NP also have been developed to improve the final step in the immune cycle, which was the process of eradication and recognition of cancer cells by cytotoxic T cells. These NPs consist of liposomes carrying IL-21 and IL-15 using maleimide groups, which successfully attached and activated T cells with cytokine secretion [167].

### 7.2. Nanoparticles Improving Immune Responses

In addition, recent studies have demonstrated that several NP-based therapies would induce an immune response or augment immune therapy through targeting TME, including photodynamic therapy (PDT), sonodynamic therapy (SDT), and photothermal therapy (PTT). In this review, we also summarized several different types of condition-sensitive linkers, which are mainly used in nano-drug delivery systems (Table 4).

(1) Photodynamic therapy (PDT) is a treatment using photosensitizers and light to absorb and convert the laser energy at the cancer site, damaging tumor cells for therapy [188]. For instance, Lu et al. developed a nanoplatform using nMOFs as strong photosensitizers for PDT, which exhibited highly effective anti-tumor efficacy [189]. These nMOFs not only promote the PDT effect without self-quenching but also allow optimization due to the structural and molecular tunability [190]. Mechanically, these nMOFs could facilitate ROS diffusion to relieve hypoxia via open channels [191]. Moreover, as a highly immunogenic therapy, PDT also could trigger ICD, recruit neutrophils, and promote T cell infiltration by acute inflammation, expression of heat-shock proteins, and the presentation of antigens [192]. Importantly, the IDOi@Hf-TBC NPs have been demonstrated to release tumor antigens and induce ICD following light irradiation in an MC38 mouse colorectal tumor model. Furthermore, the matured APCs, such as DCs and TAMs, processed and presented tumor antigens to T cells. Meanwhile, continuously released IDOi could prevent immune-suppressive metabolism to reactivate CTLs to recognize and kill cancer cells. Recently, there have been more strategies in combining PDT NPs with immunotherapy for tumor treatment. Accordingly, these PTTs combined would induce host immune responses, applied as in situ vaccines and synergize with immune checkpoint inhibitors or CAR-T to relieve therapy resistance [191].

(2) Sonodynamic therapy (SDT) could utilize sonosensitizers to generate ROS induced by US, which was applied to ablate tumors and ICD. Further synergies of SDT with immunotherapy could afford enhanced anti-cancer immunity for tumor regression. For example, Chen et al. developed an SDT NP using a manganese–protoporphyrin complex as a sonosensitizer which was loaded into liposomes and modified with FA [193]. They found that the FA-MnPs could efficiently generate oxygen even in 8 cm-deep tissues following US irradiation in a mouse model [193]. More importantly, FA-MnPs also were reported to promote the shift of TAMs from M2 to M1 type under US irradiation. Interestingly, FA-MnPs also could induce the activation of DCs, NKs, and decrease the number of Treg cells. In another study, Park et al. designed a necroptosis-inducible nanoparticle to combine SDT and anti-PD-L1 for tumor therapy [194]. The NPs consist of perfluoropentane, carboxymethyl dextran, and Ce6 as the gas precursor, hydrophilic backbone, and hydrophobic sonosensitizer [194]. Following US irradiation, the NPs could produce ROS through Ce6 and ICD of tumor cells via acoustic cavitation of perfluoropentane. They demonstrated these NP effects on immunotherapy with the maturation of DCs and activation of CD8^+^ CTLs. Interestingly, during synergy with anti-PD-L1, these NPs dramatically eliminate cancer cells in a mouse model [195]. In addition, Wang et al. designed an NP based on LA-loaded black mesoporous titania (BMT) for SDT and tumor immunotherapy [196]. Under US irradiation, the LA-BMT NP served as a sonosensitizer and NO supplementation to generate O_2_ and NO for SDT for immunotherapy [196]. The amount of O_2_ and NO cause high intracellular oxidative levels and apoptosis of tumor cells with the release of tumor-associated antigens (TAAs) [196]. The LA-BMT NPs also induced a strong immune reaction combining with anti-PD-L1 for both primary and lung distant cancer cells in a mouse model. Although the SDT NPs study provided an efficient strategy for the therapy of primary and distant cancer cells, the clinical efficacy should be improved in the future.

(3) Photothermal therapy (PTT) utilizes photothermal agents (PTAs) to kill tumor cells by converting energy of the laser into localized heat at the tumor sites [197]. Importantly, PTT NPs also could trigger ICD of tumor cells. Interestingly, hyperthermia also would cause the changes in cytokine secretion and immune reaction, which may trigger the activation of DCs and boost the transportation of chemical drugs to the lymph nodes to activate T cells [198]. For example, recent studies have reported an NP strategy using BPQDs (black phosphorus quantum dots), as an efficient PTA, encapsulated in hEX (serum exosomes). This strategy has been suggested to increase several patient-specific TAAs during hyperthermia treatment [199]. Thus, the BPQD exosomes were found to show effective outcomes by significantly increasing the temperature of the tumor site and promoting the T-cell infiltration [199]. In another study, Xu et al. developed a strategy of NPs based on the SPNE (polymeric multicellular nano-engager) for photothermal immunotherapy under NIR-II [200]. The SPNE NPs using a polymer as the photothermal core responding to NIR-II, which could accumulate on tumor sites and lymph nodes and activate the cross-interactions among tumor cells, DCs, and T cells, inducing a higher immune response [200]. Importantly, SPNE NPs triggered ICD and eliminated cancer cells via further anti-tumor immune response due to the deep-tissue penetrating NIR-II photo-irradiation. Moreover, recent studies on the immune-regulating NPs based on PTAs also suggested that more effective outcomes of PTT may be achieved by preventing the immune clearance of PTAs [201,202]. Thus, these shreds of evidence implicated that the synergistic PTT effect substantially impedes the proliferation of cancer cells and eradicates distant metastasis, as well as elevates immunological memory [203].

Accordingly, these therapies based on NPs can be incorporated with immunotherapy to improve the efficiency and specificity for drug delivery, including photodynamic therapy (PDT), sonodynamic therapy (SDT), and photothermal therapy (PTT). However, the valid outcomes still need to be tested in clinical examinations.

## 8. Using Nanoparticle-Based Drug Delivery Systems to Target Tumor Hypoxia

The tumor microenvironment is insufficient regarding oxygen provision due to the rapid growth of tumor cells and abnormal blood vasculature [95]. Insufficient oxygen caused hypoxia in TME and various events including angiogenesis, drug resistance, and metastasis. Recent studies also found that hypoxia may lead to immunotherapy resistance through activating MDSCs and Tregs, increasing CCL22-CCL28, and polarizing M 2 TAMs [204,205,206]. Hypoxia also was found to stimulate VEGF, TGF-β, and PDL-1 transcription on immune and cancer cells, resulting in immunosuppressive TME [95]. The drug resistance related to hypoxia may require O_2_ for cancer elimination in radiotherapy. Owing to the extensive tumorigenic effect of hypoxia, targeting hypoxic remains one of the main frontiers in various cancer treatment strategies. Several NP strategies were devised to target hypoxia, such as (1) delivery oxygen to hypoxia within tumor tissues by nanoparticles, (2) direct targeting of hypoxia by nanoparticles, and (3) oxygen generation in hypoxic TME using nanoparticles [94]. The following descriptions will explore some advances in this field.

### 8.1. Oxygen Delivery to Hypoxic Tumor Tissues Based on Nanoparticles

This approach may represent a promising field to replenish oxygen in the tumor microenvironment. In this case, an adequate oxygen carrier may be necessary, such as perfluorocarbon (PFC). Gao et al. utilized PFC-NPs consisting of red blood cells (RBCs) and PLGA. These PFC@PLGA-RBCM NPs showed efficient capacity for oxygen carrying and prolonged blood retention time [207]. After intravenous injection, the PFC@PLGA-RBCM NPs efficiently delivered oxygen to tumor tissues, relieved the hypoxic environment, and elevated the effect of radiotherapy. A similar strategy loading Bi_2_Se_3_-NPs with PFC also releases O_2_ by stimulation with near-infrared light [208]. Moreover, another group utilized nano-PFC oxygen shuttles to deliver oxygen to tumor issues with ultrasound power [209]. After intravenous injection, nano-PFC could rapidly release oxygen and recirculate into the lung for reoxygenation under ultrasound power. This strategy might also significantly overcome hypoxia-related drug resistance in tumor treatment. Smart NPs were developed to release oxygen depending on O_2_ demands, which were triggered by near-infrared light to achieve cooperative photodynamic or photothermal treatment [210]. These intelligent NPs achieved on-demand and continuous oxygen generation, and excellent accumulation rates and deep intratumor penetration [210]. In addition, other groups have reported decorated PEG-stabilized PFC nanoparticles. They found that the TaOX@PFC-PEG NPs could release oxygen gradually and concentrate X-rays in tumor cells using TaOx [211].

In addition, the applications of nanoparticles based on hemoglobin (Hb) have suggested a significant clinical interest, which could mimic the oxygen carrier capacity of hemoglobin, simultaneously conquering some drawbacks. The primary biological function of natural hemoglobin (Hb) is to carry and transport O_2_ between various tissues and lungs. The research efforts to develop Hb nanoparticles have used several different strategies depending on the manner of combination with Hb [212]. The first manner utilizes the polymerization of hemoglobin to inhibit its tetramer dissociation and filtration. Additionally, the second strategy focuses on incorporating anti-oxidant enzymes to prevent the switching of non-functional met-Hb. The recent research also developed a nanoplatform based on the encapsulation of Hb within a protective shell. For example, raffinose and glutaraldehyde (GA) have been applied as bi-functional cross-linking reagents to develop different poly-Hb products, such as Oxyglobin, HemoLink, and PolyHeme [213,214]. These NPs showed unacceptable toxicities. Furthermore, Lu et al. designed nanoparticles with PLGA-PEG-Hb to alleviate oxygen, which exhibited longer circulation times [214]. One of the most recent examples of the oxygen-enhanced nano-sensitizer platform was MnPcS@HPO NPs based on a combination of human serum albumin (HSA) and Hb using a disulfide relocation, which could generate oxygen and potentiate the results of sonodynamic therapy [215]. This MnPcS@HPO strategy efficiently targets cancerous niches and relieves hypoxia by harnessing the oxygen carrier capacity of hemoglobin coupled to HSA’s tumor-targeting properties [215]. These studies displayed the dramatic anti-tumor activity of sonodynamic therapy based on MnPcS@HPO in an animal model [215,216]. Another group also reported a nano-platform to alleviate hypoxia and synergize chemo-photodynamic therapy [217]. They developed an SPN-Hb@RBCM NP, which was based on the biomimetic theranostic ability of Hb, to enhance photostability, photosensitizer accumulation, O_2_ supply, and photodynamic therapy efficiency. The SPN-Hb@RBCM NP used Hb as an O_2_ transporter to reverse tumor hypoxia and re-sensitized the tumor cells to photodynamic therapy [217]. This SPN-Hb@RBCM NP provides a valuable insight into biomimetic and theranostic nanoparticles based on improving O_2_ generation and synergistic PDT/CDT treatment against tumors. Moreover, other similar NP strategies also displayed a significant effect on relieving hypoxia, such as metformin-based nanoreactors, which used methylene blue as an oxygen providing and guiding system for PDT/CDT treatment, and CeO_2_ nanocrystals anchored to MnO_2_ nanoflowers [218,219]. As these various strategies suggest, advances in nanoparticles can be harnessed to develop practical solutions that solve key issues posed by hypoxia in a tumor microenvironment. Innovative nanoparticles could deliver oxygen directly to hypoxic sites, thus, remodeling tumor environment and reactivating anti-cancer responses. In future, more studies need to focus on the translation of these theranostic solutions to clinical therapies as potential treatments in combination with chemotherapy, radio- or photodynamic therapies.

Accordingly, as these studies could efficiently deliver O_2_ to tumors this warrants further investigation to evaluate its clinical efficacy.

### 8.2. Direct Targeting of Hypoxia by Nanoparticles

Elevated ROS is known as one of cancer hallmarks. The aberrant ROS accumulation caused oxidative stress and subsequent upregulation the levels of oxidative scavengers such as glutathione [220]. Although an abnormal oxidative status promotes tumor growth, it may be utilized to potentiate therapies. An aberrant oxidative status offers an avenue for the design nanoparticles, due to the increased sensitivity of tumor cells to change the redox status [220]. For example, redox-sensitive nanoparticles could utilize the upregulated glutathione levels in cancer tissue to refine cancer therapies, which may be exploited to improve current therapeutic strategies including chemotherapy and immunotherapy [220].

There are various nanoparticles based on this strategy, so we describe main the representative approaches. (1) Liu et al. constructed GSH-sensitive NPs containing camptothecin and GalP5 [111]. These GSH-NPs can efficiently trigger drug release in TME which exhibits high GSH concentrations. Some groups developed a class of pH/GSH-sensitive NPs via self-assembly [216]. These pH/GSH-NPs displayed high stability and specificity compared to traditional photosensitizers [216]. (2) Similarly, SA-CS-NAC@IGC nanoparticles can continuously release drugs in tumor tissue under high GSH and low pH conditions [216]. After laser irradiation, these nanoparticles generated high levels of ROS, leading to the suppression of tumor growth. This class of NPs has also been utilized to construct ROS-sensitive nanomaterials for TME and re-sensitize tumor cells. Similarly, other groups developed ROS-sensitive UA NPs through joining UA molecules via ROS cleavable linkage [111]. The UA-NPs platform can selectively release chemical molecules to enhance anti-tumor effects in the stimulations of ROS.

Both ROS- and GSH-sensitive nanoparticles have been devised and tested in cooperation with current therapy. For example, some studies reported a class of GSH/ROS dual sensitive GOx@BNPs that were sensitive to elevated ROS and GSH in the tumor environment [111]. The GOx@BNPs caused an anti-cancer effect through targeting elevated ROS, low ATP, and pro-apoptotic tumor tissue [221]. PEG-PPS-GSNO@DOX-NPs were also designed to enhance drug toxicity as a dual ROS-GSH system [222]. This class of NPs displayed high NO affinity and promoted ROS-induced doxorubicin release and GSH-mediated NO release. They proved that NO reversed chemo-resistance and promoted doxorubicin accumulation without any side effects, which emphasized the promising potential of ROS/GSH dual sensitive nanomaterials for tumor therapy. As these studies suggest, these NP strategies could utilize and surmount the pathological characters of the tumor microenvironment, causing enhanced drug targeting and clinical outcomes.

### 8.3. Oxygen Generation in Hypoxic TME Using Nanoparticles

Although direct O_2_ delivery by nanoparticles is simple, the O_2_ loading capacity of NPs is limited. Another alternative strategy is to carry oxygen-producing agents, including MnO_2_, metformin, and catalase, that could generate oxygen using chemical reactions in tumor cells [111]. Based on these, some groups constructed a novel PH-H_2_O_2_ dually responsive nano-platform using albumin-decorated MnO_2_ to alleviate hypoxia [223]. This dual nano-platform synergistically enhanced the effect of both photodynamic therapy and chemotherapy through upregulating O_2_ amounts on cancer centers [223]. Li et al. developed F127@CNS-CuS/MnO_2_-NPs to enhance PTT and PDT treatments [64]. The F127@CNS-CuS/MnO_2_-NPs produced O_2_ to alleviate hypoxia in TME, further promoting therapeutic effects [64]. Furthermore, Wang et al. also designed potential NP-based MnO_2_ to boost chemo-photodynamic therapy [224]. These NPs utilized hollow polydopamine nanostructures decorated with MnO_2_ for drug delivery. Upon reaching tumor tissue by utilizing RGD, the PDA and MnO_2_ shell would release Ce6 and doxorubicin in TME. MnO_2_ can interact with H_2_O_2_ to generate O_2_, thus boosting outcomes of PDT [224]. Thus, this multifunctional nanoplatform might represent a promising approach to change hypoxia. As these studies implicated, advances in nanoparticles could be harnessed to develop clinical outcomes that address important challenges posed by the TME. These innovative nano-platforms directly deliver O_2_ to tumor tissue or cause self-generation, which would effectively remodel TME and re-sensitize chemotherapy and immunotherapy.

Other innovative technical approaches based on noble metals have also been developed to generate O_2_ in TME and enhance tumor immunotherapy, including mesoporous manganese cobalt oxide derived from MOFs [225], Pt nanoparticles decorated on MOFs [226], gold nanoclusters [227], MOF-Au NPs [228], Pd@Pt nanoplates [229], Pt-based core-shell nanoplatforms [230], Pd@Au bimetallic core-shell nanostructures [227], etc. For instance, Wei et al. have designed Pd@Pt-PEG-Ce6 NPs that could not only extend photosensitizers to tumor sites but also attract the transformation of endogenous H_2_O_2_ to O_2_ over a long period of time [229]. They also found that the moderate photothermal character of Pd@Pt-PEG-Ce6 could promote the process of decomposition following 808 nm laser irradiation. Moreover, Yang et al. developed a Pd@Au core-shell nanostructure NP that would continuously catalyze the decomposition of cellular H_2_O_2_ to O_2_, which effectively relieves hypoxia and restores the resistance [227]. Under the irradiation of an NIR-II laser, the catalytic efficiency of Pd@Au NPs has been enhanced via the plasmon resonance effect. In addition, Liu et al. designed AuNCs-NH_2_ NPs to produce O_2_ to improve photodynamic therapy, which was encapsulated with PAMAM [227]. Importantly, these NPs displayed the catalase-like activity from pH 4.8 to 7.4 [227]. Tao et al. reported multifunctional HABT-C NPs exhibiting multienzyme activities for sonodynamic therapy to alleviate hypoxia and inhibit immune suppression. These NPs could produce O_2_ via a cascade reaction and amplify redox signaling to promote apoptosis of tumor cells [231]. Importantly, TiO_2_ could facilitate oxidative injury and ROS production in tumor cells [231]. Actually, HABT-C@HA NP exhibited satisfactory anti-cancer activity in animal experiments [231].

## 9. Discussion

Cancer cells have developed various properties that differentiate them from normal cells. These properties are known as hallmarks such as active proliferation, angiogenesis, genomic instability, invasion and metastasis, inflammation, immune evasion, epigenetic reprogramming, and phenotypic plasticity [3,232]. Traditional therapies including surgery, radiotherapy, and chemotherapy, are standard in tumor clinical treatment; however, the resistance of these therapies remains as the main barrier due to the hallmarks of cancer. Thus, exploring alternative treatment options has been demonstrated to be an important strategy. Recently, tumor immunotherapy has been demonstrated to be a powerful and novel therapeutic regimen with great durability and specificity against cancers [5]. However, current cancer immunotherapy is limited by barriers such as low tumor specificity, poor response rate, and systemic toxicities, which result in the development of primary, adaptive, or acquired resistance. The rate of proliferation and migration exceeds the rate of carcinoma cell removal by the immune system, so it cannot be eliminated by the immune systems. Moreover, immunotherapy resistance has complex mechanisms that depend on the interaction between tumor cells and the tumor microenvironment (TME). Therefore, targeting TME has recently received attention as a promising approach for re-sensitizing resistant neo-plastic niches to existing cancer immunotherapy. Cancer immunologists are focus on elevating the effectiveness of cancer treatment by reversing or neutralizing the immune evasion mechanism of these cancer cells. Hence, a better understanding of tumor microenvironment dependencies in specific tumor tissues is key to defining the aspects of TME that caused resistance; only then can vulnerabilities be explored to provide better cancer treatments in the near future.

With the development of nanotechnology, nano-platforms possess outstanding features, including high loading capacity, tunable porosity, and specifically targeted to the desired locus. Therefore, nanoplatforms can significantly improve the effectiveness of immunotherapy while reducing its toxic and side effects on non-target cells that receive intense attention in cancer immunotherapy. With these porous properties, NPs can load a large number of biomolecules, deliver them in a targeted manner, modulate the TME, and regulate the immune systems. In this review, we summarized novel nanoparticle strategies targeting the tumor microenvironment reprogramming enhancing immunotherapy, such as targeting macrophages, targeting fibroblasts, targeting vasculature, targeting hypoxia, and oxidative stress. Although a great change has been undertaken to facilitate the advances in NPs for immune therapy, the applications of nanoparticles in clinical trials still remain in a fledging period. With interdisciplinary synergism and accumulated science findings, inspiringly, the process will be accelerated with expected breakthroughs in nanoplatforms for cancer treatment. Nanoplatforms are promising and will play an increasingly significant role in the field of tumor treatment.

## Figures and Tables

**Table 1:** Various TME components for immunotherapy resistance focused on in this review.

Types	Main Mechanisms	References
CAFs (cancer-associated fibroblasts)	CAFs were proved to be associated with cancer therapy resistance by secreting chemokines, metabolites, and growth factors, such as interleukin-17A (IL-17A), interleukin-6 and interleukin-8, ELF, FGF5, HGF, STC1, IGFBP3, and TGF-β2.	[32,33,34,35]
Immune cells	Various immune cells have been reported to promote an immunosuppressive TME for therapy resistance, which mainly including effector and regulatory T cells, cytotoxic T cells (CTLs), tumor-associated macrophages (TAMs), myeloid-derived suppressor cells (MDSCs), etc.	[36,37,38]
ECM (extracellular matrix)	The ECM was found to provide structural support and regulate cellular activities, including proliferation, communication and adhesion, including laminin, elastin, and collagen. In general, the ECM contributes to tumor therapy resistance through promoting the escape from immune surveillance and inhibiting drug delivery.	[39,40,41,42]
Vasculature	Recent studies have demonstrated that the outcome of tumor treatment is influenced by the vasculature through drug delivery and the supply of nutrients and O_2_. Additionally, the large bio-molecules were significantly impeded and transported far away tumor tissue. In addition, the accumulation of metabolic waste and an insufficient amount of O_2_ promote acidic and hypoxic environments, which contributes to drug resistance.	[43,44]
Hypoxia	The aberrant vasculature and excessive requirement for O_2_ of tumor cells may create a hypoxic tumor microenvironment. Hypoxia could activate HIF-1 to promote tumor cell proliferation, adapt to hypoxia, and become resistant to various therapies. In addition, hypoxia upregulates P-glycoprotein and dihydrofolate reductase, which contributes to the topoisomerase II targeting drug resistance.	[19,44,45,46,47,48,49]

**Table 2:** Advantages or disadvantages of the main nanoparticle types mentioned in this review.

Types	Inorganic NPs	Organic NPs	Hybrid NPs	Ref.
Typical NPs	Mesoporous NPs	COFs	MOFs	[68]
Advantages	Good biocompatibility High drug loading capacity Optical physicochemical properties High catalytic properties	Improved biocompatibility Biodegradability Controllable particle size Different functionalization	Good biocompatibility Biosensing High catalytic activity Optical properties	[69,70]
Disadvantages	Poor biodegradability Potential toxicity	Limited pore size Degradability	Potential toxicity Limited pore size Degradability	[71]

**Table 3:** The main characters of nanoparticles mentioned in this review, were summarized.

Mechanisms for Elevating Immunotherapy	Composition of NPs	PNMs	Target Cells	Main Results	Ref.
Enhancing uptake and presentation	PMSN@OVA-MPN	PMSN	DCs	PMSN@OVA-MPN prevented cancer cell proliferation and enhanced immune response	[72]
	UiO-OVA	Zircoium-based nMOF	APCs	UiO-OVA produce forceful antigens and effectively triggered CTLs	[73]
	W-TBP/CpG/PD-L1	Castionic nMOF	DCs	W-TBP NPs promoted antigen presentation	[74]
	LPSiNPs	PSi	B cells	LPSiNPs enhanced the activation of APCs and B cells	[75]
	IMHCS-OVA	IMHCSs	APCs	IMHCS-OVA promote the maturation of APCs	[76]
Tumor-targeted delivery	PSiPs-HER2	PSiNP	Cancer cells	PSiPs-HER2 achieved specific targeting and destruction of tumor cells	[77]
	MSN@polyphenol	MSN	Cancer cells	MSN@polyphenol achieved controlled molecule release	[78]
	CpG/ZANPs	MOFs	APCs	CpG/ZANPs targeted lymph nodes, and APCs, significantly inhibiting tumor proliferation	[79]
	CD@MSNs (carbon nanodots-based MSNs)	MSNs	TAMs, NKs	CD@MSNs combined with PTT could accumulate in the tumor and eliminated cancer cell metastasis	[80]
	LCP-II NPs	Calcium phosphate NPs	Cancer cells	The LCP-II NPs delivered drugs to tumor sites in a xenograft model	[81]
	PHNPs@DPA-S-S-BSA-MA@3-MA	PHNPs	TAMs	PHNPs exhibited efficiency for targeting TAMs, enhancing immune reaction, and preventing cancer development	[82]
Reversing immunosuppressive TME	Fe_3_O_4_-OVA nano-composites	Fe_3_O_4_ nanoparticles	TAMs, BMDC	The NPs stimulated the maturation of BMDCs and the activation TAMs to prevent cancer progress and development	[83]
	OX/IND-MSNP	MSNPs	APCs, cancer cells	The OX/IND-MSNP combined with immunotherapy leading to ICD and immune suppressive effects	[84]
	MIL-100 with MTO, hyaluronic acid	MOF (MIL-100)	Cancer cells	The NPs induced ICD and reversed the immunosuppressive effects	[85]
	IMD@Hf-DBP/αCD47	nMOFs	TAMs, cancer cells	Under X-ray irradiation, the NPs reversed the immunosuppressive effects	[86]
	ZIF-8/CpG ODNs	ZIF-8 NPs	TAMs	The NPs showed less cytotoxicity and enhanced the uptake of CpG ODNs in TAMs, and increased the levels of cytokines	[87]
	Ce6/MLT@SAB	Hybrid NPs	Cancer cells	The NPs combined with PDT further upregulated the level of CD4 + and CD8 + T cells in tumor sites and reduced the numbers of MDSCs	[88]
Multi-functionality	IMD@Hf-DBP/αCD47	nMOFs	TAMs, cancer cells	The NPs enhanced systematic immune responses through the combination of RT-RDT	[86]
	Cu-TBP	Cuporphyrin nMOF	Cancer cells	Cu-TBP elicited systemic anti-cancer immune responses by activating immune responses in primary and metastatic tumors	[89]
	MOF-OVA@CpG	MOF	APCs	Co-delivery of antigen and CpG triggered T cell activation and cytokine secretion, and inhibited cancer development	[90]
	COF-609	COF	Cancer cells	The study offered the first integration of PDT and immunotherapy by 3D COFs to inhibit cancer metastasis and recurrence and demonstrated a new way to design ICD inducers	[91]
	COF@ICG@OVA	COF	DCs	The NPs combined with NIR irradiation and a checkpoint inhibitor inhibited cancer progress and development	[92]
	FeSe_2_-PE	FeSe_2_ nanoflower	Cancer cells	The FeSe_2_-PE-NPs were fabricated to achieve the on-demand release of H_2_Se on NIR-II photoactivation to kill tumor cells	[67]
	H-MnO_2_-PEG/C&D	Mesoporous MnO_2_ nanoshells	Cancer cells	The NPs as a multifunctional theranostic platform regulated TME and PTT/PDT therapy and enhanced immunotherapy	[93]

**Table 4:** Different types of condition-sensitive linkers, which are mainly used in nano-drug delivery systems.

Type	Compounds	Chemical Formula	References
pH sensitive	cis-aconityl derivatives	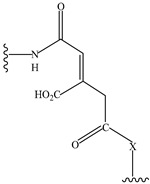	[168,169,170]
	Orthoesters	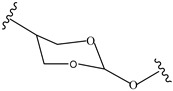	[171,172]
	N-ethoxybenzylimidazoles	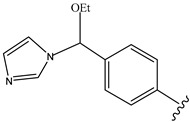	[173,174]
	Silyl ethers	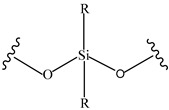	[175]
	Imine derivatives	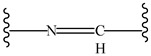	[176]
	Β-thiopropionate	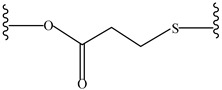	[177]
	Vinylethers	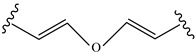	[178]
	Hydrazine derivatives	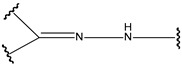	[179]
	Acetal and ketal derivatives	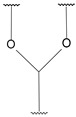	[180]
ROS sensitive	Disulfides	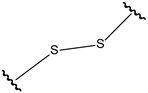	[181]
	Diselenide	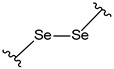	[182]
Enzyme sensitive	Cathespin B sensitive	Gly-Pro-Ile-Cys (Et)-Phe-Phe-Arg-Leu-Gly-Lys (FITC)-Cys-NH	[183]
	Thrombin sensitive	Gly-(D)Phe-Pro-Arg-Gly-Phe-Pro-Ala-Gly-Gly	[184]
	MMP	Phe-Lys-Gly	[185]
Hypoxia responsive	Nitroaromatic derivatives (destabilizer, radiosynthesizer)	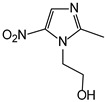	[186]
	Azobenzenes	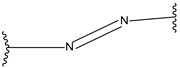	[187]

## Data Availability

No new data were used.
